# Probiotics as Microbiome Modulators in Male Infertility: Rethinking Dysbiosis Across the Gut–Testis Axis

**DOI:** 10.3390/jpm16020099

**Published:** 2026-02-06

**Authors:** Aris Kaltsas, Spyros Pournaras, Ilias Giannakodimos, Eleftheria Markou, Marios Stavropoulos, Stamatis Papaharitou, Fotios Dimitriadis, Athanasios Zachariou, Nikolaos Sofikitis, Michael Chrisofos

**Affiliations:** 1 Third Department of Urology, Attikon University Hospital, School of Medicine, National and Kapodistrian University of Athens, 12462 Athens, Greece; ares-kaltsas@hotmail.com (A.K.); iliasgiannakodimos@gmail.com (I.G.); stamarios@yahoo.gr (M.S.); 2 Laboratory of Clinical Microbiology, Attikon University Hospital, School of Medicine, National and Kapodistrian University of Athens, 12462 Athens, Greece; spournaras@med.uoa.gr; 3 Department of Microbiology, University Hospital of Ioannina, 45500 Ioannina, Greece; eleftheria.markou4@gmail.com; 4 Andrology Laboratory and Sperm Bank, 54622 Thessaloniki, Greece; sdrcauth@gmail.com; 5 Department of Urology, Faculty of Medicine, School of Health Sciences, Aristotle University of Thessaloniki, 54124 Thessaloniki, Greece; helabio@yahoo.gr; 6 Laboratory of Spermatology, Department of Urology, Faculty of Medicine, School of Health Sciences, University of Ioannina, 45110 Ioannina, Greece; azachariou@uoi.gr (A.Z.); nsofikit@uoi.gr (N.S.)

**Keywords:** male infertility, dysbiosis, gut–testis axis, androbactome, oxidative stress, probiotics, synbiotics, microbiome

## Abstract

Male infertility contributes substantially to couple infertility, and a large proportion of cases remain idiopathic. Dysbiosis within the gut, seminal, and urinary microbiomes has been associated with impaired semen parameters, reproductive tract inflammation, and oxidative stress. This narrative review, informed by a structured literature search, summarizes current evidence for the gut–testis axis and the androbactome in male infertility and discusses mechanistic pathways linking microbial imbalance to sperm dysfunction. Proposed mechanisms include immune activation, increased oxidative stress, endocrine and metabolic perturbations, and disruption of epithelial barriers, including the blood–testis barrier. Early clinical trials report that selected probiotic or synbiotic formulations may be associated with improvements in one or more World Health Organization (WHO) semen parameters and with reductions in oxidative or inflammatory biomarkers (surrogate laboratory endpoints; pregnancy and live-birth outcomes are rarely reported and remain unproven) in selected populations, such as idiopathic infertility and the post-varicocelectomy setting. Given patient heterogeneity, a personalized approach requires prespecified clinical phenotypes and measurable monitoring targets, rather than indiscriminate supplementation. At present, probiotics should be considered an adjunct rather than a stand-alone therapy. Well-designed, contamination-aware microbiome studies and adequately powered randomized trials with clinically meaningful endpoints, including pregnancy and live birth, are required before routine clinical implementation. This synthesis is intended to support personalized counseling and trial design by clarifying candidate phenotypes, appropriate monitoring endpoints, and realistic limitations of current evidence.

## 1. Introduction

Infertility affects a substantial proportion of reproductive-age couples, and male factors contribute to approximately 30% to 50% of cases [1]. Declines in semen parameters have been reported over recent decades [2]. A large meta-analysis estimated a marked reduction in sperm concentration from 1973 to 2011 [3], and subsequent analyses suggest that declines may extend beyond Western regions [4]. Proposed contributors include cardiometabolic and lifestyle factors, environmental exposures, oxidative stress, and heat exposure, which may act synergistically [5,6,7]. Population-based studies also link modifiable exposures—including higher body mass index (BMI) and recreational drug use—with poorer semen quality [8,9]. These trends strengthen the rationale for earlier, prevention-oriented counseling and individualized risk modification alongside standard etiologic evaluation. Despite standard evaluation, approximately 30% of men with abnormal semen parameters have no identifiable cause, supporting the need for additional modifiable targets and precision approaches [10].

The human microbiome is a potential modifiable factor in male reproductive health. Microorganisms in the intestine and urogenital tract produce metabolites and interact with immune, endocrine, and metabolic networks that may influence testicular function [11]. The gut–testis axis describes bidirectional signaling, through which gut microbial composition and microbial products may affect spermatogenesis and endocrine regulation [12]. In this review, microbiome refers to the community of microorganisms and their collective genetic potential within a defined niche, as characterized by culture-based or culture-independent methods. Dysbiosis denotes a context-dependent disruption in community structure or function associated with an adverse host phenotype, and taxonomic differences alone do not establish pathology or causation [13]. The male reproductive tract is a low-biomass environment in which microbial DNA can be detected in semen and urine, and the term androbactome is used here as an umbrella for these microbial signatures without implying viable colonization of the testis [14]. For clarity, the seminal microbiome refers specifically to microbes detected in semen. Because semen and urine are low-biomass niches, sequencing-based results require contamination-aware interpretation supported by appropriate negative controls, standardized sampling and abstinence conditions, and transparent reporting of biomass or read depth and decontamination workflows [15]. Without such precautions, spurious signals from contamination could be mistaken for true microbiome findings, severely limiting their interpretability.

Evidence increasingly links dysbiosis in the gut, semen, and urinary tract with abnormal semen parameters and adverse reproductive outcomes [13]. Proposed mechanisms include persistent inflammation and oxidative stress, altered epithelial and barrier integrity, and endocrine disruption, each of which may impair spermatogenesis and sperm function [16]. Interventional studies evaluating probiotics and synbiotics have reported improvements in selected semen parameters and oxidative biomarkers in some settings, although findings remain inconsistent [17,18,19].

This review synthesizes evidence across microbial niches relevant to male infertility and integrates mechanistic and clinical data on microbiome modulation. Emphasis is placed on human studies, including randomized trials and robust observational cohorts, while preclinical evidence is used to support biological plausibility. Practical considerations for patient selection, formulation, dosing, and outcome monitoring are discussed, alongside methodological standards for low-biomass reproductive microbiome research. By integrating evidence across the gut–testis axis and the male reproductive tract microbiome with a focused appraisal of probiotic and synbiotic interventions, this review frames the field through a personalized medicine lens, distinguishing what is supported by human data, identifying candidate endotypes for intervention, and outlining decision-oriented endpoints for testing and monitoring. Most available evidence remains associative, and the clinical efficacy of microbiome-directed interventions remains unproven without adequately powered randomized trials that include pregnancy and live-birth endpoints.

## 2. Methods and Literature Search Strategy

**Figure 1:**
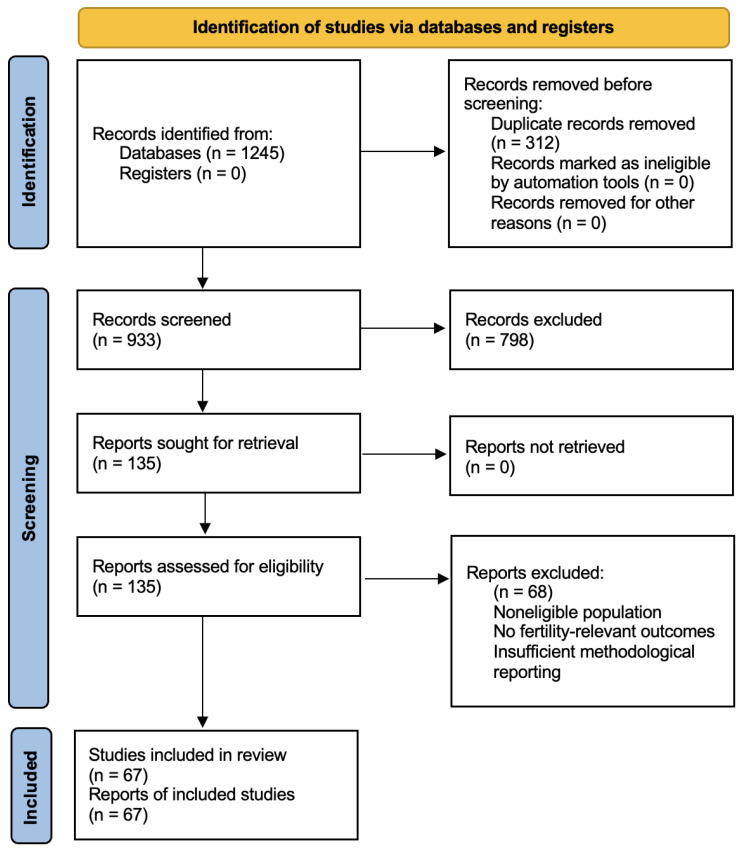
PRISMA 2020 flow diagram of database search, screening, and inclusion.

A structured literature search was performed to enhance transparency and reproducibility. Although this work is a narrative review (qualitative synthesis), the literature identification and study selection processes were reported in accordance with the PRISMA 2020 statement [20]. The review protocol was not registered. A completed PRISMA 2020 checklist (Supplementary Material File S1) and a PRISMA flow diagram are provided (Figure 1).

PubMed/MEDLINE, Embase, Web of Science Core Collection, and the Cochrane Library (CENTRAL) were searched from database inception to 31 December 2025. Search concepts combined terms for male infertility and semen quality with terms for microbiome composition or dysbiosis across gut and male urogenital niches, together with terms for microbiome-modulating interventions, including probiotics, synbiotics, and prebiotics. No lower date limits were applied. Complete database-specific search strings, limits, and yields are reported in Table 1.

Searches were conducted from database inception to 31 December 2025. Complete, database-adapted strategies are reported below, including controlled vocabulary where applicable and free-text terms. Search strings are shown as executed.

Eligibility criteria were predefined. Included records comprised English-language full-text publications in adult men relevant to fertility, encompassing randomized controlled trials, nonrandomized interventional studies, and observational designs including cohort, case–control, and cross-sectional studies. Systematic reviews and major clinical guidelines were also screened to identify additional eligible primary studies and to contextualize the evidence base. Eligible studies were required to report at least one fertility-relevant endpoint, including conventional semen analysis parameters, sperm DNA fragmentation indices, oxidative stress or redox biomarkers, inflammatory markers, reproductive hormones, or clinical reproductive outcomes such as pregnancy, time to pregnancy, or live birth. Excluded records comprised non-English publications, conference abstracts without full text, editorials, and single-patient case reports. Animal and in vitro studies were not considered primary evidence and were included only when they contributed to mechanistic plausibility not otherwise available from human studies. Studies without extractable fertility-relevant outcomes or without sufficient methodological detail to permit appraisal were excluded. When multiple reports described overlapping cohorts, the most complete or most recent report was retained.

Search results were imported into a reference manager and de-duplicated. A total of 312 duplicates were removed, yielding 933 unique records for screening. Title and abstract screening was conducted independently by two reviewers. Following screening, 798 records were excluded as not meeting eligibility criteria. The remaining 135 articles underwent full-text assessment by two reviewers, with discrepancies resolved through discussion and consensus and, when required, arbitration by a third reviewer. Sixty-seven studies met eligibility criteria and were included in the narrative synthesis. Sixty-eight full-text articles were excluded for reasons including non-eligible population, absence of fertility-relevant outcomes, or insufficient methodological reporting.

Data extraction captured study design, population characteristics, microbiome niche and methods, intervention characteristics where applicable (strain composition, dose, and duration), and fertility-related outcomes, including semen parameters, sperm DNA integrity measures, oxidative stress or redox biomarkers, and clinical reproductive outcomes. Given heterogeneity in study designs, interventions, and outcome definitions, evidence was synthesized qualitatively rather than pooled quantitatively. Intermediate laboratory outcomes were interpreted as surrogate signals and were not assumed to translate into pregnancy or live-birth benefits in the absence of direct evidence.

Study quality and rigor were appraised using a qualitative, domain-based framework. Randomized trials were assessed using Cochrane RoB 2 domains, including randomization, deviations from intended interventions, missing outcome data, outcome measurement, and selective reporting. Observational studies were evaluated for selection bias, phenotype and outcome definitions, confounding control, and laboratory and analytical rigor relevant to microbiome research. Because semen and urine are low-biomass specimens, semen and urine microbiome studies were specifically assessed for contamination-control reporting, including the use of negative controls and blanks, handling of batch effects, and reporting of microbial biomass proxies or sequencing depth and decontamination workflows. Domain-level judgments are summarized in Table 2 to enhance transparency and to provide a concise overview of study limitations across designs. These elements were incorporated into the interpretive weighting of individual studies within the qualitative synthesis, and studies were not excluded solely on the basis of appraisal; rather, methodological limitations were used to down-weight confidence in findings where appropriate.

## 3. Microbial Dysbiosis in Male Infertility

Microbial dysbiosis refers to disruption of a microbial ecosystem, often characterized by reduced beneficial taxa and expansion of opportunistic organisms [21]. In male infertility, dysbiosis can involve multiple interconnected niches, including the gut, semen, and urinary tract [21]. However, dysbiosis is highly context-dependent. Functional alterations in microbial communities and their metabolites, rather than taxonomic shifts alone, determine its impact. Accordingly, compositional changes should not be interpreted as evidence of causality.

### 3.1. Gut Microbiome

The gut microbiome modulates metabolic, immune, and endocrine pathways relevant to male reproductive function [22]. The GELDING (Gut Endotoxin Leading to a Decline in Gonadal Function) hypothesis proposes that diet- or antibiotic-associated dysbiosis increases intestinal permeability and endotoxin translocation, driving low-grade inflammation that can suppress Leydig cell steroidogenesis and disrupt hypothalamic–pituitary–gonadal signaling [23]. In experimental models, high-fat diets and endotoxin exposure have been associated with reduced testosterone and impaired sperm parameters, including reduced motility and increased abnormal morphology [24,25]. These findings support biological plausibility for a gut–testis axis but do not establish clinical efficacy of microbiome-directed interventions in humans.

Human studies also suggest an association between gut microbial composition and semen phenotypes. In a 2023 cross-sectional study that profiled gut, semen, and urine microbiota across men with different semen abnormalities, gut microbial alpha diversity differed from urine and semen and specific taxa differed across semen phenotypes [26]. Genetic epidemiology provides additional support. A Mendelian randomization study identified several gut genera that were positively associated with male infertility risk, including *Allisonella*, *Anaerotruncus*, *Barnesiella*, *Intestinibacter,* and *Lactococcus*, whereas *Bacteroides*, *Romboutsia*, and some *Ruminococcaceae* were inversely associated [27]. These observations are hypothesis-generating and require confirmation in longitudinal cohorts and interventional trials.

More recent Mendelian randomization analyses have expanded these findings by examining specific male reproductive phenotypes. A 2025 study reported causal associations between specific gut genera and male infertility, orchitis and epididymitis, abnormal spermatozoa, and sperm-related proteins, and also reported potential associations with erectile dysfunction [28]. Mendelian randomization can reduce confounding, but inference depends on instrument validity and is vulnerable to horizontal pleiotropy and weak-instrument bias. Across the gut literature, associations should be interpreted cautiously because observational datasets remain susceptible to residual confounding from diet, adiposity, smoking, and recent antibiotic exposure, and cross-sectional designs cannot establish temporality or exclude reverse causality. Geographic and population-level variation in baseline microbiota further constrains generalizability across cohorts [29]. In addition, key mechanistic insights supporting the gut–testis axis are derived primarily from animal and other preclinical models, and several proposed pathways, including those inferred from diet-induced dysbiosis and fecal microbiota transplantation paradigms, lack consistent corroboration in prospective human studies and interventional trials.

### 3.2. Seminal Microbiome

Semen contains microbial DNA and can harbor bacteria from the urethra and accessory glands. Culture-independent sequencing has identified a diverse semen microbiome, although results vary across studies because of low biomass, sampling conditions, and contamination risk [30,31]. A 2021 systematic review and meta-analysis reported that Lactobacillus was associated with better semen parameters, whereas *Prevotella*, *Ureaplasma urealyticum*, *Enterococcus faecalis*, and *Mycoplasma hominis* were associated with poorer semen parameters and higher DNA fragmentation index [32]. These associations do not establish causality, and the potential for reverse causation and confounding remains substantial.

Case–control and cohort studies suggest that semen microbiome profiles differ between infertile phenotypes and fertile controls. In a pilot next-generation sequencing study, men with nonobstructive azoospermia had a distinct seminal microbiome compared with fertile controls [33]. In a cross-sectional cohort study, abnormal sperm motility was associated with a higher abundance of *Lactobacillus iners*, and abnormal sperm concentration was associated with differences in several Pseudomonas species [34]. A larger 2025 study reported that seminal communities could cluster into genus-dominant profiles, and specific taxa were associated with sperm functional parameters [35]. However, not all cohorts demonstrate clear separation between fertile and infertile men, and reported associations vary in magnitude, underscoring biological and methodological heterogeneity.

Interpretation of semen microbiome studies is complicated by methodological and clinical heterogeneity. Abstinence interval, recent antibiotics or probiotics, sexual activity, and comorbidities can influence semen microbial profiles and may confound associations with semen parameters [31]. Many studies rely on 16S ribosomal ribonucleic acid (16S rRNA) sequencing or targeted polymerase chain reaction (PCR) and include limited reporting of negative controls, making contamination and batch effects important sources of bias in low-biomass samples, thereby limiting confidence in such findings. Standardized sampling protocols, reagent controls, and reporting of bacterial load are required to improve reproducibility and clinical interpretability. Differences in DNA extraction methods, targeted 16S rRNA regions, sequencing platforms, and bioinformatic pipelines may further contribute to inconsistent findings across studies.

Given the low microbial biomass of semen, even minimal background DNA introduced during collection, extraction, or library preparation can disproportionately influence taxonomic profiles. Contamination-aware workflows are therefore essential and should include extraction blanks, reagent-only controls, and negative controls processed in parallel, together with reporting of bacterial load to support the discrimination of biologic signal from the laboratory background [36]. Without these measures, reported microbiome profiles may largely reflect contaminants rather than true microbiota, severely limiting the validity of such studies. Standardization of sampling conditions, particularly the ejaculatory abstinence interval and recent exposures that may alter microbial profiles, is also required to reduce avoidable variability and improve between-study comparability. In addition, reliance on 16S rRNA sequencing limits taxonomic resolution and often precludes strain-level inference, which is relevant because strains within the same species may differ in virulence, inflammatory potential, or metabolic activity. These methodological constraints likely contribute to the heterogeneity and occasional inconsistency of associations reported between individual taxa and semen quality across cohorts.

At present, routine diagnostic testing of the seminal microbiome in asymptomatic infertile men is not supported by sufficient evidence [36]. Microbiome profiling remains primarily a research tool, and the clinical utility of taxonomy-based signatures will depend on validated links to outcomes and on whether targeted modulation improves fertility endpoints. Future work should distinguish commensal dysbiosis from overt genital tract infection and should incorporate female partner and couple-level outcomes.

### 3.3. Urogenital (Urinary and Reproductive Tract) Microbiome

Beyond the gut and semen, microbial communities are also detected in the male urogenital tract, including urine, the urethra, and accessory glands [37]. Culture-independent methods have revealed low-biomass yet consistent microbial signatures in urine, even among asymptomatic individuals, supporting the concept of a male urinary microbiome [38]. Associations between urine microbiome composition and semen analysis parameters have been reported, although causality remains uncertain [39]. Co-sampling of urine and semen requires rigorous negative controls and contamination-aware workflows to convincingly distinguish biological overlap from shared sources and laboratory contamination [34,39].

Emerging evidence links urinary tract microbiome imbalances with male subfertility. A recent study by Osadchiy et al. found that certain urinary microbial profiles correlate with abnormal semen parameters, suggesting that urine microbes might reflect underlying issues in sperm development [39]. For example, men with poor sperm motility had a nearly 50-fold higher urinary abundance of *Dialister micraerophilus* compared to men with normal motility, while those with low sperm counts showed significantly lower levels of *Enterococcus faecalis* in their urine [39]. These findings provide preliminary evidence illustrating that specific bacteria in urine associate with impaired sperm motility or concentration. This is consistent with the notion that subclinical colonization or dysbiosis in the urinary tract may be linked to reduced sperm quality.

Emerging evidence suggests that bladder and prostatic dysbiosis may contribute to chronic, often subclinical, inflammation within the male genital tract, potentially harming sperm during transit and maturation [40]. Even in the absence of overt infection, a low-grade inflammatory milieu, characterized by leukocytes, pro-inflammatory cytokines, and excess reactive oxygen species (ROS), can impair spermatogenesis, disrupt accessory-gland function, and degrade semen quality [41]. Consistent with this, men with chronic prostatitis or recurrent urinary tract infections frequently exhibit reduced fertility, attributable in part to inflammation-mediated injury to sperm and accessory glands [41].

Established genital tract infections remain clinically relevant. Sexually transmitted and uropathogenic bacteria such as *Chlamydia trachomatis*, *Neisseria gonorrhoeae*, *Mycoplasma species*, and *Escherichia coli* can cause epididymitis, urethritis, and prostatitis, which can lead to scarring or obstruction and impair sperm transport [42]. These infections can also provoke marked local inflammation and oxidative stress that reduce sperm viability [43]. Beyond overt infection, urogenital dysbiosis may contribute to a subclinical inflammatory environment that is unfavorable for sperm maturation and function [13].

Overall, low-biomass urinary and seminal microbiome profiles have been linked to semen phenotypes, supporting the concept that local dysbiosis may sustain low-grade inflammation detrimental to sperm maturation and function [34,39].

Several confounders should be explicitly considered when interpreting urinary microbiome studies in male infertility. Sexual activity and exposure to a partner’s genital microbiota can alter the male urethral microbial profile and may influence urinary signals [44]. The interval since last ejaculation may also affect microbial detection and relative abundance. Recent antibiotic or probiotic exposure, lower urinary tract symptoms, chronic prostatitis phenotypes, and systemic comorbidities such as obesity, diabetes, or immune-mediated disorders can further modify urinary microbial communities and bias comparisons between fertile and infertile groups. Methodological factors are equally important, including collection approach and timing, urine fraction captured, and laboratory batch effects introduced during DNA extraction, sequencing, and bioinformatic processing. Most reported associations between urinary taxa and semen parameters remain correlational and may reflect inflammation, sampling conditions, or shared risk factors rather than a stable resident community that directly contributes to impaired fertility.

## 4. Mechanisms: How Microbial Dysbiosis Affects Male Fertility

**Figure 2:**
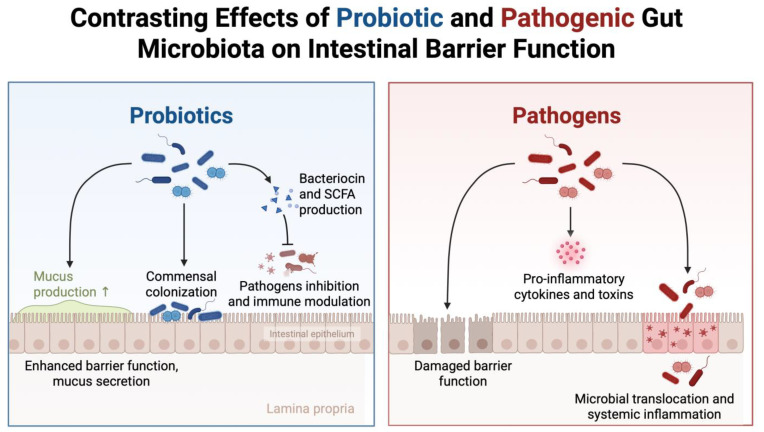
Schematic representation of the contrasting effects of probiotic and pathogenic gut microbiota on intestinal barrier function. Probiotics promote eubiosis, reinforce epithelial tight junctions, and produce short-chain fatty acids (SCFAs) that enhance immune homeostasis and limit pathogen overgrowth and endotoxin translocation. Conversely, pathogenic bacteria disrupt barrier integrity, release pro-inflammatory cytokines and toxins, and promote microbial translocation and systemic inflammation. These barrier-level mechanisms underlie the gut–testis axis discussed in the text. Created in BioRender. Kaltsas, A. (2025) (https://BioRender.com/3n2mvja, accessed on 1 January 2026).

Several mechanisms have been proposed to link microbiome dysbiosis with impaired male fertility, but the supporting evidence is uneven across pathways. To minimize overinterpretation, human observational/interventional findings are distinguished from animal-model and in vitro/ex vivo mechanistic evidence throughout this section; where direct human data are lacking, pathways are presented as biological plausibility rather than established clinical effects. Inflammation and oxidative stress have the most consistent human backing, largely through clinical associations between leukocytospermia or elevated seminal reactive oxygen species and poorer semen quality. By contrast, endocrine findings in men are variable and typically modest, and most detailed endocrine mechanisms derive from animal and in vitro studies rather than clinical cohorts. Proposed epigenetic effects and disruption of blood–testis barrier integrity are supported predominantly by animal and ex vivo data, with limited direct validation in humans [45]. Other candidate pathways, including metabolite-driven effects on sperm energetics and hypotheses about intergenerational transmission, remain early-stage and should be interpreted cautiously because human evidence is sparse. These mechanisms are not independent. Inflammatory signaling can intensify oxidative injury, and both can secondarily influence endocrine regulation and testicular microenvironments. Figure 2 summarizes the barrier-centered framework in which microbial balance shapes epithelial integrity and immune activation along the gut–testis axis.

### 4.1. Inflammation and Immune Dysregulation

One of the most consistently supported links in human studies between dysbiosis and infertility is through chronic inflammation. Microbial imbalances, whether in the gut or genital tract, can sustain inflammatory signaling. Human clinical studies link male infertility with a systemic inflammatory profile, with higher circulating pro-inflammatory cytokines interleukin (IL)-1β, interleukin (IL)-6, and tumor necrosis factor (TNF)-α alongside increased oxidative stress markers that correlate with poorer semen quality and higher sperm DNA fragmentation [46]. In contrast, most testis-level immune mechanisms are derived from animal and in vitro work, showing that inflammatory signaling can disrupt blood––testis barrier (BTB) integrity and activate innate immune pathways such as Toll-like receptor signaling (TLR), thereby reducing immune privilege and impairing spermatogenesis [47].

In the male reproductive tract, bacteriospermia and related microbial patterns are associated with leukocytospermia and higher concentrations of pro-inflammatory cytokines in seminal fluid [48]. These immune factors can impair sperm function: cytokines and other inflammatory mediators in semen are associated with reduced sperm motility and can induce sperm apoptosis or morphological defects [49]. Bacteria themselves may release endotoxins and proteins that damage the delicate sperm cells [50]. For example, *Escherichia coli* in semen can attach to sperm cells and agglutinate them or cause tail abnormalities; other bacteria produce enzymes and toxins that injure the sperm membrane and acrosome [50,51].

Local inflammation can also alter the male reproductive tract environment in more structural ways. Chronic prostatitis or seminal vesicle inflammation can lead to fibrosis or partial blockages in the ducts, impairing sperm transport and ejaculation [52]. Inflammation of the testicular tissue or epididymis, often due to infections, can damage the spermatogenic epithelium or disrupt sperm maturation, respectively. Even low-grade inflammation without obvious infection could disrupt the BTB or create an autoimmune scenario where sperm antigens are exposed to the immune system [53,54]. Indeed, the seminal microbiome is thought to play a role in sperm antigenicity and immune tolerance. Dysbiosis might break the normal immune tolerance to sperm, increasing the risk of anti-sperm antibodies that impair fertilization. There is also evidence that the male and female reproductive microbiomes interact; microbes from the male mic can influence female tract immunity [55]. An imbalanced seminal microbiome in the male could provoke female immune responses after intercourse, potentially affecting embryo implantation or pregnancy maintenance [15]. In short, dysbiosis can tip the immune balance from a tolerogenic, sperm-friendly state to a hostile, inflamed state, thereby reducing fertility [15].

At the molecular level, in experimental and ex vivo systems, lipopolysaccharide (LPS) activates Toll-like receptor 4 (TLR4)–myeloid differentiation primary response 88 (MyD88)–IκB kinase (IKK)–nuclear factor κB (NF-κB) signaling, amplifying cytokine production and leukocyte recruitment within the male tract; in these models, chronic activation can impair Leydig-cell steroidogenesis and damage sperm DNA [19]. Moreover, microbiota-driven IL-6 signaling perturbs Sertoli-cell junctional dynamics, priming BTB leakiness and immune exposure of germ cells [19].

### 4.2. Oxidative Stress

Oxidative stress is closely intertwined with inflammation in the context of infertility. Sperm cells are uniquely vulnerable to oxidative damage—their plasma membranes are rich in polyunsaturated fatty acids, making them prime targets for lipid peroxidation, and have limited internal antioxidant capacity [56]. A dysbiotic microbiome can exacerbate oxidative stress in several ways. First, activated immune cells generate ROS as a defense mechanism [56]. Excess ROS in seminal fluid can attack sperm membranes and DNA, decrease motility and increase DNA fragmentation rates [56]. Clinical studies consistently find that men with infertility (especially those with infection or high semen white cell counts) have elevated ROS levels and oxidative damage markers in semen [57]. For instance, bacteriospermia is associated with significantly higher ROS generation in semen compared to sterile samples [58]. Imbalanced microbial communities may also deplete antioxidant factors in seminal plasma or produce metabolites that further propagate free radicals [57].

A 2024 systematic review (JBRA Assisted Reproduction) outlines multiple, complementary antioxidant mechanisms through which probiotics may counteract seminal redox imbalance, ranging from direct radical scavenging and metal-ion chelation to upregulation of host antioxidant defense and reinforcement of gut barrier integrity that limits endotoxin translocation [59]. Consistent with these mechanisms, human trials commonly report increases in total antioxidant capacity (TAC) and reductions in malondialdehyde (MDA) in semen, often in parallel with improvements in standard semen parameters [59]. In addition, trials have also documented decreased seminal hydrogen peroxide (H_2_O_2_) levels and a lower sperm DNA fragmentation index (DFI) following probiotic supplementation, further connecting oxidative stress mitigation to these laboratory endpoints [18,60]. Importantly, such measures are regarded as intermediate surrogate outcomes of improved semen oxidative status rather than direct clinical fertility endpoints like pregnancy or live birth [61].

In preclinical models, gut dysbiosis amplifies systemic oxidative stress: translocation of lipopolysaccharide and other microbial products activates inflammatory and redox pathways within reproductive tissues, including the testes [62]. In rodent models, high-fat-diet-induced dysbiosis elevates testicular ROS and compromises germ-cell development [63], whereas microbiome correction, via fecal microbiota transplantation or targeted dietary oligosaccharides, raises testicular antioxidant enzyme activity, lowers ROS, restores spermatogenesis, and improves sperm quality [64,65,66]. Collectively, these results suggest that dysbiosis can indirectly induce oxidative damage in the testes, compounding local ROS sources, and support mechanistic plausibility for the existence of a causal gut–testis axis [65,66]. These preclinical interventions primarily support mechanistic plausibility; translation to improved human fecundity requires confirmation in well-controlled clinical studies.

Overall, excess ROS associated with dysbiosis can lead to peroxidative damage of the sperm cell membrane, oxidative DNA damage in sperm (contributing to infertility and miscarriage risk), and even apoptosis of spermatozoa or germ cells [45]. Mechanistically, this is one of the best-understood pathways linking infection/inflammation to male infertility and likely extends to non-infectious dysbiosis as well [45]. Encouragingly, many probiotics are known to have antioxidant properties or to induce the host’s antioxidant defenses, which may counteract this mechanism. In humans, improvements in oxidative-stress biomarkers (e.g., higher TAC and lower MDA or hydrogen peroxide, H_2_O_2_) are encouraging surrogate signals, but direct evidence that these changes translate into higher pregnancy or live-birth rates remains limited.

Beyond spermatogenic injury, microbiome-related oxidative stress may also impair erectile physiology. Excess ROS quenches bioavailable endothelial nitric oxide (NO), disrupts nitrergic signaling within cavernosal tissue (“ROS-NO uncoupling”), and thereby compromises erection quality [67]. This link to erectile dysfunction (ED) is clinically important because ED contributes to infertility indirectly: even with preserved spermatogenesis, ED can lower natural fecundity by reducing intercourse frequency, penetration adequacy, and effective intravaginal semen deposition [67]. Accordingly, ED should be viewed as an intercourse-mediated barrier to conception rather than a primary testicular defect, highlighting that microbiome-driven oxidative stress may influence fertility via both direct effects on sperm and indirect effects through impaired erectile function.

### 4.3. Endocrine and Hormonal Disruption

Microbiome dysbiosis may affect male fertility by perturbing endocrine pathways that regulate spermatogenesis and sexual function [68]. The gut microbiome can influence systemic hormones through metabolic effects and microbial metabolite signaling [69]. However, evidence linking gut microbiome features to circulating reproductive hormones in humans remains limited and heterogeneous [70]. When differences are observed, they are often modest and may be confounded by adiposity, diet, comorbidities, and medication exposure [71]. Thus, endocrine disruption should currently be considered a plausible mechanism rather than a consistently demonstrated clinical phenotype in men [72].

Dysbiosis is associated with obesity and metabolic syndrome, which are linked to lower testosterone and higher estradiol in men [73]. Whether microbiome alterations contribute independently to these hormonal patterns or largely reflect shared metabolic risk remains uncertain. Short-chain fatty acids (SCFAs) produced from dietary fiber can influence enteroendocrine signaling, including glucagon-like peptide 1 (GLP-1) [74]. In clinical settings, GLP-1 receptor agonists have been reported in some studies to increase testosterone and improved sperm parameters in men with metabolic disease [75,76,77]. This supports a pathway through which reduced production of beneficial metabolites in dysbiosis may adversely affect the hypothalamic–pituitary–gonadal (HPG) axis, but direct causal evidence in humans is not yet established [78].

Additional mechanistic support comes from a 2023 preclinical restraint-stress model in which oral *Lactobacillus* (with or without a prebiotic) preserved serum testosterone and testicular histology by upregulating steroidogenic enzymes and antioxidant defense while reducing pro-inflammatory cytokine expression. Collectively, supplementation attenuated hypothalamic–pituitary–adrenal axis (HPA axis) activation and protected spermatogenesis under stress [79]. While these findings support biological plausibility, translation to human endocrine outcomes requires confirmation.

Microbiota can influence sex steroid metabolism through enzymes such as beta-glucuronidases and hydroxysteroid dehydrogenases, which affect deconjugation and recycling of steroids [80,81]. Dysbiosis may therefore contribute to subtle endocrine perturbations relevant to spermatogenesis, although the magnitude and direction of these effects in vivo remain unclear. In animal models, supplementation with Lactobacillus strains has been associated with preservation of testosterone levels in aging mice [82], whereas antibiotic-mediated microbiome disruption has been linked to reduced testosterone and impaired sperm production [83]. These findings support biological plausibility but do not establish clinical benefit in humans.

On the flip side, male sex hormones themselves can shape the microbiome—androgen deprivation therapy in men leads to shifts in gut microbiota composition. This bidirectional interaction complicates the picture but underscores that the microbiome and hormones are tightly interlinked [84]. In parallel, endocrine-metabolic perturbations linked to dysbiosis also manifest clinically as ED via gut–brain–hormone interactions and endothelial dysfunction. The narrative synthesis therefore proposes microbiome-targeted adjuncts (probiotics/prebiotics, dietary modulation, fecal microbiota transplantation) in selected men with ED although the current evidence base is preliminary [85].

### 4.4. Other Pathways: Epigenetic and Barrier Effects, Intergenerational Transmission

Beyond the above three pillars, additional layers (supported predominantly by animal, in vitro, or ex vivo data and therefore hypothesis-generating in humans) may link the microbiome to male reproductive potential.

#### 4.4.1. Sperm DNA Integrity and Epigenetics

Beyond oxidative-stress-mediated DNA fragmentation, it has been hypothesized that dysbiosis could influence sperm epigenetic regulation, but direct human evidence linking microbiome features to sperm epigenetic marks is limited, and most mechanistic support comes from animal or ex vivo studies. One-carbon metabolism and methylation capacity are shaped by gut microbes through the production of B-vitamins and folate; commensals also contribute to methionine cycling and the generation of S-adenosylmethionine (SAM), the universal methyl donor, and produce short-chain fatty acids that influence histone acetylation [86]. A dysbiotic gut may therefore theoretically reduce methyl-donor availability or increase toxic intermediates such as homocysteine, leading to abnormal DNA or histone methylation in germ cells. In human studies, aberrant sperm DNA methylation and impaired chromatin packaging have been associated with infertility and adverse embryo development [86].

Animal studies further indicate that the paternal diet and microbiome composition can remodel the composition of sperm small non-coding RNAs (sncRNAs). In male mice, a high-fat diet increases specific transfer-RNA (tRNA) fragments and PIWI-interacting RNAs (piRNAs) in sperm. Microinjection of these tRNA fragments into control zygotes partially recapitulates metabolic phenotypes in offspring [87]. It should be noted that aberrant sperm DNA methylation and chromatin packaging defects have been observed in infertile men (human studies), whereas alterations in the sperm sncRNA payload and their phenotypic effects on offspring have thus far been demonstrated only in animal models [88,89]. Collectively, these observations are consistent with a model in which chronic microbiome-related stressors, via inflammation and oxidative stress, leave an epigenetic imprint on sperm (such as altered sncRNA payload or DNA methylation patterns) that can compromise fertility and potentially transmit risk to the next generation. However, whether dysbiosis directly drives these epigenetic changes in men, as well as whether any such changes translate into intergenerational effects in humans, remains uncertain.

#### 4.4.2. Blood–Testis Barrier Integrity

The BTB is a physical and immunological barrier formed by tight junctions between Sertoli cells, crucial for providing a protected environment for germ-cell development. Systemic inflammation and endotoxemia arising from gut dysbiosis can disrupt these junctional complexes in rodent models. In particular, intraperitoneal LPS lowers testosterone, impairs spermatogenesis, increases oxidative stress, and downregulates occludin, claudins, and other BTB proteins, thereby compromising barrier integrity [90]. If gut-derived endotoxins from dysbiosis chronically leak into the circulation, they could similarly weaken the BTB over time, as shown in these models [90]. Direct evidence that BTB weakening occurs in humans remains limited, largely because noninvasive biomarkers that capture BTB functional integrity have not been established in clinical cohorts [91]. Validation of this mechanism in men would require the demonstration of plausible surrogate indicators of barrier compromise, such as the presence of antisperm antibodies or the detection of tight-junction-associated proteins in semen, together with evidence that these findings track with chronic endotoxemia or systemic inflammatory burden [92]. A compromised BTB may permit immune-cell infiltration or antibody access to developing germ cells and facilitate entry of toxins into the testicular microenvironment, ultimately reducing sperm output. This mechanism plausibly contributes to the well-described association between chronic inflammatory disorders and impaired spermatogenesis.

#### 4.4.3. Paternal Microbiome and Offspring Health

Preconception paternal microbiome status has been proposed as a contributor to offspring health and development. In animal models, paternal gut microbiome perturbation before mating has been associated with adverse offspring outcomes that can be mitigated by microbiome restoration [93]. Mechanistic studies have implicated changes in sperm small RNAs and metabolite profiles. Human evidence remains limited. However, current human data are insufficient for any clinical recommendation; this remains hypothesis-generating. Paternal nutrition and lifestyle factors, including diet and adiposity, have been linked to offspring outcomes through pathways that include microbiome-associated metabolism and epigenetic regulation [94]. These data are hypothesis-generating and do not support clinical recommendations for microbiome modulation to improve offspring outcomes.

#### 4.4.4. Energy Metabolism and Neuromodulation

Sperm motility and capacitation are highly adenosine triphosphate-dependent processes supported by coordinated glycolysis in the principal piece and mitochondrial oxidative phosphorylation in the midpiece [95]. During capacitation, sperm increases glucose uptake and metabolic flux, enabling downstream endpoints, including hyperactivated motility [96]. Disruption of glucose transport impairs sperm bioenergetics and function. In mice, inhibition or genetic deletion of the sodium glucose cotransporter 1 (SGLT 1) reduces adenosine triphosphate production and compromises motility and fertilizing capacity [97,98].

Microbiome-derived metabolites provide plausible upstream inputs into these energy pathways. SCFAs can act as substrates and signaling ligands. In an obese mouse model, butyrate supplementation improved sperm parameters and partially normalized the testicular metabolomic profile [99]. In human ex vivo experiments, propionate activated the sperm olfactory receptor 51E2 (OR51E2) and induced calcium, and cyclic adenosine monophosphate linked signaling with kinematic changes consistent with capacitation [100]. Microbiota-shaped bile acids and tryptophan-derived indoles represent additional metabolic and neuroactive routes. Bile acid excess impaired male fertility in mice through Takeda G protein-coupled receptor 5 (TGR5)-related mechanisms [101], whereas farnesoid X receptor (FXR) is detectable in human sperm and bile acid or FXR agonism modified motility and capacitation in vitro [102]. Indole 3 propionic acid can enhance mitochondrial respiration through peroxisome proliferator-activated receptor beta delta-dependent metabolic programs [103], and indole derivatives improved the cation channel of sperm (CatSper)-linked motility in rodent injury models [104]. Overall, aside from limited ex vivo human sperm observations for SCFA OR51E2 and bile acid FXR signaling, most evidence supporting a microbiome-metabolite-to-bioenergetics-to-motility axis remains preclinical and should be interpreted as biological plausibility rather than direct clinical proof.

**Figure 3:**
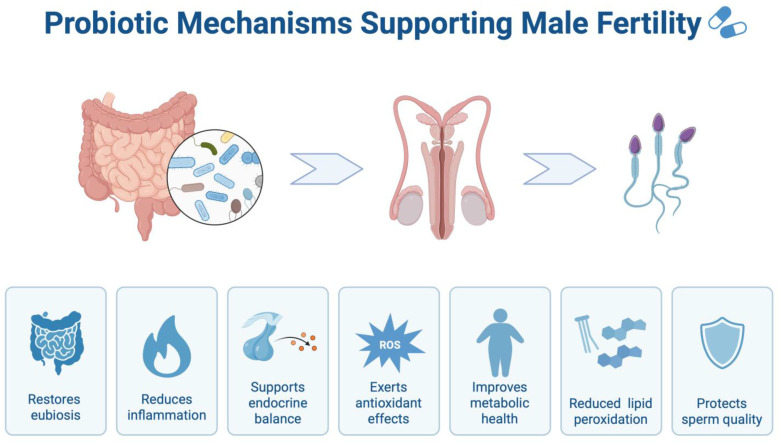
Probiotic mechanisms supporting male fertility. Schematic of the gut–testis axis summarizing the principal actions of probiotics relevant to male reproduction: restoration of eubiosis; reduction in inflammation and oxidative stress; modulation of endocrine–metabolic crosstalk; and a potential net improvement in sperm quality. The term “androbactome” is used here as an umbrella for microbial communities across the male genital tract, whereas the seminal microbiome denotes the ejaculate-level community detected in semen; the schematic is not intended to imply a resident testicular microbiome. Abbreviations: TAC, total antioxidant capacity; MDA, malondialdehyde; H_2_O_2_, hydrogen peroxide; ROS, reactive oxygen species. Created in BioRender. Kaltsas, A. (2025) (https://BioRender.com/qqps44u, accessed on 1 January 2026).

Figure 3 summarizes the mechanistic framework that links probiotic use to the observed clinical signals. Importantly, semen parameters and oxidative-stress biomarkers are intermediate endpoints, and the current interventional literature provides limited direct evidence for improved time to pregnancy, pregnancy rates, or live-birth outcomes.

## 5. Clinical Evidence for Probiotics and Synbiotics

Given the mechanistic implications between microbiome imbalance and male infertility, an important question is whether correcting dysbiosis can improve male reproductive outcomes. Probiotics, defined as live beneficial microorganisms, and synbiotics, which combine probiotics with prebiotic substrates, have been investigated as strategies to restore eubiosis and counter the inflammatory and oxidative milieu associated with impaired spermatogenesis [105]. This section summarizes the current clinical evidence, beginning with guideline statements, followed by data from human trials and reviews, and concluding with mechanistic considerations consistent with observed clinical effects. The rationale is that dysbiosis amplifies inflammatory and oxidative pathways that damage sperm and perturb endocrine crosstalk; re-establishing eubiosis may therefore be beneficial.

### 5.1. Emerging Recognition in Guidelines

The 2025 European Association of Urology (EAU) Guidelines on Sexual and Reproductive Health include, for the first time, a qualified mention of probiotics as a potential adjunctive therapy [106]. This cautious statement, not a strong recommendation, reflects small randomized controlled trials reporting improvements in at least one World Health Organization (WHO) semen parameter and, in some studies, reductions in sperm DNA fragmentation. However, the Guidelines emphasize that the certainty of evidence is low and that larger, well-designed trials are required before routine adoption [106].

Accordingly, probiotics are considered only as a complement in selected scenarios (e.g., idiopathic male factor infertility or the early post-varicocelectomy period) and should not replace established treatments such as varicocele repair, antimicrobial therapy for infection, or endocrine management [106]. In practice, clinicians should interpret this guideline language to mean that probiotic therapy is an adjunctive, phenotype-guided option and not a substitute for standard etiologic evaluation or treatment. This position places microbiome-targeted interventions on the clinical radar while underscoring the need for more robust evidence.

### 5.2. Human Trials and Reviews

Randomized trials in idiopathic male factor infertility suggest that selected synbiotic or probiotic formulations may improve intermediate laboratory endpoints (e.g., semen analysis parameters and oxidative stress markers), but no significant effects on pregnancy or live-birth rates have been demonstrated to date. In a triple-blind RCT (*n* = 56), 80 days of the multi-strain synbiotic FamiLact (*Lactobacillus*/*Bifidobacterium*/*Streptococcus* with fructo-oligosaccharides) improved sperm concentration, motility, and morphology and reduced lipid peroxidation and sperm DNA fragmentation compared with placebo [18]. In a separate double-blind RCT (*n* = 52), a multi-strain probiotic administered for 10 weeks increased semen volume, total sperm number, concentration, and motility, alongside higher TAC and lower MDA and inflammatory markers, with minimal sex-steroid changes [107].

More recently, a double-blind RCT in idiopathic oligoasthenoteratozoospermia (OAT) (*n* = 73) evaluated synbiotic supplementation as an adjunct to an antioxidant formulation. Compared with antioxidant plus placebo, antioxidant plus FamiLact yielded greater improvements in progressive motility and immotile sperm percentage and larger reductions in sperm DNA fragmentation after three months [108].

Supportive, hypothesis-generating data include a double-blind placebo-controlled pilot study (*n* = 41) using a synbiotic containing Lactobacillus paracasei with prebiotic components for 24 weeks, which reported improvements across multiple semen parameters and modest increases in gonadotropins and testosterone [109]. A randomized crossover study in asthenozoospermic donors (*n* = 9) found that 6 weeks of *Lactobacillus rhamnosus* CECT8361 plus *Bifidobacterium longum* CECT7347 increased motility and reduced intracellular hydrogen peroxide and DNA fragmentation, with attenuation after washout [60].

In the postoperative setting, a double-blind RCT after microsurgical varicocelectomy (*n* = 78) reported that three months of FamiLact was associated with higher sperm concentration and normal morphology at 3-month follow-up compared with placebo, with non-significant trends in motility and volume [17].

A 2024 systematic review restricted to randomized controlled trials (RCTs) concluded that probiotic or synbiotic supplementation improved at least one WHO semen parameter and at least one oxidative-stress marker in each included trial but emphasized small samples, short intervention windows, and substantial heterogeneity in strain composition and dosing [59]. Pregnancy and live-birth outcomes were infrequently captured, and trials were not powered for these endpoints, limiting inferences about couple-level fecundity [59]. Contemporary narrative and scoping reviews similarly highlight multi-strain *Lactobacillus*/*Bifidobacterium* preparations administered for 8–12 weeks as the most consistently associated with improvements in semen and redox endpoints, with a favorable safety profile [110,111,112].

The critical appraisal and risk of bias are as follows. Although the available studies are largely described as double- or triple-blind randomized trials, the overall body of evidence is of low certainty and is best interpreted as having a substantial risk of bias and imprecision. Most trials enrolled small cohorts (typically <100 participants; in one case, single-digit sample size) and were conducted in single centers, limiting statistical power, increasing the probability of chance findings, and reducing external validity. Key design and reporting elements that influence internal validity—such as sequence generation, allocation concealment, adherence assessment, handling of missing data, and pre-specified outcomes/analysis plans—are variably detailed across reports, raising concerns about selective reporting and multiplicity when numerous semen and biomarker endpoints are tested. Several interventions also involve proprietary, branded multi-strain formulations; where funding sources and manufacturer involvement are incompletely or inconsistently reported, a potential risk of sponsorship bias cannot be excluded. In addition, follow-up is generally short (weeks to a few months), populations are heterogeneous (idiopathic infertility/OAT spectrum, post-varicocelectomy cohorts), and co-interventions (e.g., concurrent antioxidants in both arms) complicate the attribution of effects to the microbiome-targeted agent alone [108]. Finally, potentially influential confounders—including diet and lifestyle patterns, duration of ejaculatory abstinence, and recent/intercurrent antibiotic exposure—have not been consistently measured or controlled, and most studies prioritize surrogate laboratory endpoints rather than pregnancy or live-birth outcomes, for which they are not powered [59]. Collectively, these limitations necessitate cautious interpretation: observed improvements in semen/redox markers should be viewed as hypothesis-generating until confirmed in larger, multicenter, independently funded, prospectively registered RCTs powered for clinically meaningful fertility outcomes [59].

To facilitate comparison across randomized studies, the principal interventional trials are summarized in Table 3.

### 5.3. Mechanistic Rationale Consistent with Clinical Signals

Improvements in TAC together with reductions in MDA and lower sperm DNA fragmentation align most directly with redox pathway modulation, consistent with attenuation of lipid peroxidation and oxidative DNA injury as proximate drivers of better motility and sperm quality [19,59]. Where decreases in inflammatory markers or leukocytospermia accompanied semen improvements, the signal is most compatible with an immune pathway, in which reduced inflammatory activation limits leukocyte-mediated oxidative stress within the male reproductive tract [107,109,113,114]. By contrast, sex steroid changes were inconsistent across studies, suggesting that any endocrine effects are likely secondary rather than the dominant mechanism in the current clinical evidence [109]. Claims of a consistent reshaping of the ejaculate microbiome remain preliminary and require strain-resolved confirmation embedded within randomized trials that also measure microbial metabolites alongside clinical endpoints [14]. Table 4 summarizes the directionality of these signals across trials and emphasizes that these are intermediate laboratory endpoints, since pregnancy and live birth outcomes have not been captured.

## 6. Practical Clinical Considerations

Interest in probiotic therapy for male infertility is increasing, yet use should remain cautious and adjunctive. The aim in clinical practice is to identify men most likely to benefit, select an appropriate formulation and duration, and monitor for objective change while maintaining standard care.

### 6.1. Candidate Patients

Potential candidates include men with idiopathic male factor infertility, particularly those with a measurable redox or inflammatory phenotype. Examples include unexplained OAT with an elevated sperm DNA fragmentation index [60]. Men who continue to exhibit suboptimal semen parameters after successful varicocelectomy (i.e., in the post-varicocelectomy period) may also be considered [17]. In men with obesity or metabolic syndrome, conditions frequently accompanied by dysbiosis, a structured weight-reduction program, with or without probiotic supplementation, may support both metabolic and reproductive health [115]. A recent meta-analysis reported significant increases in sperm concentration and progressive motility, together with reductions in the sperm DNA-fragmentation index, following weight loss [116].

Beyond specific medical interventions, systematic attention to modifiable lifestyle determinants is essential in prevention oriented counseling for male infertility. In a cross-sectional fertility clinic population, a substantial proportion of men were overweight or obese. Although the association between body mass index and conventional semen parameters is not uniformly observed across studies, excess adiposity is consistently linked to lower testosterone concentrations and broader endocrine dysregulation that can undermine male reproductive potential [9]. Recreational drug use has also been associated with adverse seminal characteristics, including lower semen pH, and an inverse relationship has been reported between body mass index and sperm progressive motility [8]. Collectively, these observations support early counseling that prioritizes achievable lifestyle targets such as weight optimization, healthy dietary patterns, and avoidance of tobacco and recreational drugs, with moderation of alcohol intake where relevant, as part of comprehensive infertility care. Men with clearly defined, non-microbiome etiologies, such as obstructive azoospermia, hypogonadotrophic hypogonadism requiring gonadotrophins, or genetic azoospermia, are unlikely to derive fertility-specific benefit from probiotics, although general health effects remain possible. Because fertility is couple-mediated, any adjunctive probiotic strategy should be positioned within a couple-level plan and should not delay time-sensitive escalation to assisted reproductive technology (ART) when indicated.

### 6.2. Formulations and Dosing

Most positive clinical signals have been observed with multi-strain formulations containing *Lactobacillus* and *Bifidobacterium*, often paired with a prebiotic fiber. A minimum course of 8–12 weeks is reasonable [59]. Administration with food may improve gastrointestinal survival, and storage should follow manufacturer guidance to preserve viability [117]. Patients should select reputable preparations with specific strain designations (genus–species–strain ID) and stated potency at the end of shelf life [118]. Where possible, it is preferable that products to be used contain strains mirroring those evaluated in clinical trials (e.g., *Lactobacillus rhamnosus*, *Bifidobacterium longum*, *Lactobacillus paracasei*; multi-strain mixes similar to study products such as FamiLact or Flortec), at daily doses on the order of 10^9^–10^10^ CFU/day for 8–12 weeks [59,60].

### 6.3. Safety and Contraindications

Probiotics are generally well tolerated in immunocompetent adults. Transient, self-limiting gastrointestinal symptoms, such as bloating, flatulence, or diarrhea, are the most commonly reported and typically occur early in therapy [119]. These mild effects usually resolve on their own and do not require medical intervention [119].

Serious adverse events are rare but have been described predominantly in high-risk patients. In certain high-risk scenarios such as severe immunosuppression, critical illness, or the presence of an indwelling central venous catheter, cases of probiotic-associated bacteremia or fungemia have been reported [120]. Given these rare but important risks, probiotic use should be avoided in such patients, and careful patient selection is advised. Similarly, probiotics are contraindicated in acute severe pancreatitis, where some trials have reported worse outcomes with probiotic therapy [120]. Beyond these documented complications, theoretical risks include systemic infection, deleterious metabolic effects such as D-lactic acidosis, and exaggerated immune activation in susceptible individuals, although these events appear to be very uncommon [121].

For most patients, the benefit–risk profile is favorable when probiotics are used as adjuncts to standard care. Nevertheless, improvements in semen parameters do not guarantee natural conception, particularly when a female factor coexists, so expectations should remain realistic. Across the randomized infertility trials summarized in this review, tolerability was generally comparable to placebo, and no significant or serious adverse effects were reported. The overall tolerability in these studies was comparable to that of controls, reinforcing that probiotics were safe in the studied populations.

### 6.4. Integration with Established Care

Probiotics should be positioned as adjunctive measures within standard male infertility care and should not replace etiologic evaluation or established treatments. Consideration may be reasonable in men with idiopathic infertility or a phenotype consistent with inflammatory or oxidative dysregulation, particularly when conventional management and lifestyle optimization have been initiated. Proven genitourinary infections require targeted antimicrobial treatment, and established indications for varicocele repair, endocrine therapy, or assisted reproduction should not be deferred. In men with leukocytospermia or suspected genital tract inflammation, short courses of anti-inflammatory or antimicrobial therapy may be appropriate, with subsequent probiotic supplementation considered as supportive care for microbiome restoration [122]. In a cohort of infertile men with male accessory gland infection, combining antibiotics with a probiotic formulation was associated with higher bacterial eradication rates compared with antibiotics alone, supporting adjunct use in selected patients after appropriate pathogen-directed therapy [123].

Probiotic supplementation should not be initiated as monotherapy in men with suspected or confirmed genitourinary infection, persistent symptoms, or recent antibiotic exposure without documentation of eradication. Use should be deferred until appropriate cultures, targeted antimicrobials, and clinical resolution have been achieved. When a specific, treatable etiology is established, management should prioritize definitive interventions and avoid framing probiotics as an alternative to causal therapy. In couples in whom assisted reproduction is indicated because of severe male factor infertility or limited female reproductive time, probiotics may be continued as supportive care but should not extend the interval to IVF or ICSI [11]. Coadministration during antimicrobial therapy should be considered only when compatible with the chosen regimen and when safety criteria are met.

Integration should follow a time-limited framework aligned with spermatogenesis. Lifestyle optimization remains foundational and includes a Mediterranean-style dietary pattern, regular physical activity, smoking cessation, reduced alcohol intake, adequate sleep, and avoidance of excessive scrotal heat exposure. A pragmatic approach is to implement lifestyle measures with adjunct probiotic supplementation and reassess objective endpoints after one spermatogenic cycle, typically about three months, using repeat semen analysis and additional biomarkers where available. Male infertility can also signal broader health vulnerability, with severity-graded associations reported for all-cause mortality. Routine care should include cardiometabolic risk assessment, endocrine evaluation, mental health support, and targeted oncologic surveillance when clinically indicated [124].

### 6.5. Monitoring and Outcomes

Objective reassessment after approximately three months is advisable. A repeat semen analysis should document concentration, progressive motility, and morphology. When baseline sperm DNA fragmentation is elevated, repeat testing is useful to evaluate whether DNA integrity has improved. Where available, oxidative stress assessment can complement semen analysis, with the measurement of total antioxidant capacity and malondialdehyde providing clinically interpretable indices of redox balance. Randomized trials have reported concurrent improvements in semen parameters with favorable changes in total antioxidant capacity, malondialdehyde, and sperm DNA fragmentation, supporting their role as monitoring endpoints in selected patients [59,107].

Hormonal reassessment is not routinely required and should be reserved for men with baseline endocrine abnormalities or new symptoms suggesting hormonal dysfunction. Pregnancies occurring during or after the intervention period should be recorded, while avoiding causal attribution at the individual level [107].

Crucially, improvements in semen parameters, sperm DNA fragmentation, or redox biomarkers represent intermediate laboratory outcomes and should not be presented as evidence of improved couple-level fecundity. Pregnancy, live birth, and time to pregnancy are inconsistently captured in probiotic and synbiotic trials, and changes in surrogate endpoints have not been shown to reliably translate into higher pregnancy or live birth rates [125]. These measures are therefore best used to guide time-limited, individualized decision-making, including predefined criteria for continuation or discontinuation and timely progression to established fertility interventions when indicated.

### 6.6. Decision Points

Probiotic supplementation, when used, should be delivered within a predefined therapeutic window and evaluated against objective stopping rules. Since the interval from spermatogonial differentiation to mature spermatozoa, together with epididymal transit, approximates three months in men, reassessment after three to four months is biologically coherent [126]. Discontinuation is appropriate when follow-up testing shows no clinically meaningful improvement in pre-specified endpoints such as WHO semen parameters and, where measured, sperm DNA fragmentation or oxidative stress indices, because the available clinical trial literature is short and does not support open-ended continuation [59]. Therapy should also be stopped if clinically relevant adverse effects develop or tolerability is poor, recognizing that reported events in adults are usually mild and gastrointestinal, while rare invasive infections have been described mainly in high-risk settings [121]. Probiotic use should not delay etiologic management or time-sensitive escalation to assisted reproductive technology ART, including in vitro fertilization IVF or intracytoplasmic sperm injection ICSI, when indicated. When objective improvement is documented and the preparation remains well tolerated, a further limited extension of three to six months may be considered, provided that progression to definitive interventions remains timely and is not deferred.

## 7. Future Directions

Research on the microbiome–male-infertility interface remains at an early stage of development. The current evidence base is constrained by predominantly cross-sectional or single-center designs, limited mechanistic resolution, and inconsistent reporting of standardized clinically meaningful outcomes. Progress will depend on adequately powered and methodologically rigorous studies, complementary experimental models that clarify causal pathways, and trial frameworks anchored to patient-relevant endpoints such as pregnancy and live birth. The following subsections begin with specific limitations identified in the preceding sections and propose targeted solutions in terms of study design, endpoints, and control measures.

### 7.1. Couple-Based Longitudinal Studies

Most available studies profile only one partner and rely on a single sampling time point, leaving the within-couple microbial exchange largely unresolved. This limitation complicates the interpretation of shared taxa, since apparent overlap may reflect transient transfer, specimen contamination, or laboratory artifacts rather than stable colonization. It also weakens causal inference because microbiome variation cannot be aligned with the timing of conception attempts or subsequent fertility outcomes [127].

Progress requires prospective longitudinal cohort designs at the couple level with coordinated sampling from both partners across relevant anatomical sites, including stool, vaginal and cervical compartments, semen, and urine, with endometrial sampling reserved for clearly justified clinical contexts. Sampling should be repeated across menstrual cycles and aligned with periods of intercourse to capture temporal patterns of acquisition and loss and to determine whether specific microbial configurations persist during windows that are biologically relevant to conception. Clinical endpoints should be embedded from the outset, with time to pregnancy and assisted reproduction outcomes collected using prespecified definitions, so that microbial dynamics can be evaluated against outcomes that carry direct clinical meaning. Cross-sectional observations of limited inter-partner transmission in infertile couples further support the need for repeated measures that can distinguish stable sharing from short-lived exchange and clarify whether couple-specific microbiome states contribute to reduced fecundability [128].

### 7.2. High-Resolution Multi-Omics and Host Integration

The current evidence is heavily shaped by reliance on 16S rRNA sequencing, which offers limited taxonomic granularity and infers function only indirectly. This constraint limits identification of the specific organisms and pathways most plausibly linked to sperm dysfunction and reduces the ability to separate biologically meaningful signals from background variation. In parallel, many studies do not adequately account for host-level modifiers such as genetic variation, metabolic state, inflammatory burden, and oxidative stress, all of which can influence both microbiome composition and reproductive phenotypes and may therefore confound observed associations [129].

Advancement in this area depends on higher-resolution multi-omics paired with systematic host integration. Shotgun metagenomics and metatranscriptomics can resolve organisms at the species or strain level and quantify functional potential and activity, while targeted metabolomics can connect microbial outputs to host inflammatory profiles, redox balance, and semen quality measures. Microbiome datasets should be analyzed alongside host- and semen-derived layers that capture relevant biology, including semen proteomics, oxidative stress indices, sperm DNA fragmentation, and clinically meaningful endocrine and metabolic parameters, supported where feasible by host genotyping. Such integration offers a clearer framework for mechanistic inference by linking microbial functions to measurable downstream pathways rather than relying on taxonomic associations alone. Genetic epidemiology approaches such as Mendelian randomization may further support causal inference by leveraging host variants associated with microbial traits, but any signals derived from these methods require confirmation in prospective cohorts and interventional studies designed to test whether modifying a candidate pathway alters reproductive outcomes [130].

### 7.3. Interventional Trial Design and Precision Approaches

Interventional evidence remains constrained by small sample sizes, single-center conduct, and limited statistical power, with substantial heterogeneity in interventions, treatment duration, and reported endpoints. Many trials focus on intermediate laboratory outcomes and rarely capture pregnancy or live birth, which limits clinical interpretability and leaves uncertainty regarding which microbiome-directed therapies meaningfully improve fertility. Inconsistent outcome definitions and reporting further impede cross-trial comparison and synthesis [59].

Progress requires the prioritization of multicenter randomized controlled trials with adequate enrolment and prespecified core outcome sets. Trials should assess microbiome-directed interventions for efficacy and safety using harmonized semen endpoints alongside validated measures of oxidative stress and inflammation, while also incorporating couple-level outcomes, including time to pregnancy, clinical pregnancy, and live birth. The adoption of standardized reproductive endpoints will enable meaningful aggregation of evidence and reduce selective reporting. Factorial or pragmatic designs can evaluate whether microbiome modulation provides incremental benefit when combined with established lifestyle interventions such as dietary optimization or structured weight loss. Precision elements should be built into the design through stratification or planned subgroup analyses based on baseline oxidative stress, sperm DNA fragmentation, metabolic phenotype, and a reproducible dysbiosis metric, thereby testing whether treatment effects are concentrated in biologically defined subgroups. Large, well-governed trials with robust outcome capture are essential to determine whether microbiome modulation translates from changes in laboratory measures to improvements in pregnancy and live birth [59].

### 7.4. Methodological Rigor in Low-Biomass Studies

Low-biomass sampling in semen and testicular tissue creates a substantial vulnerability to contamination, because exogenous DNA from reagents, consumables, and the laboratory environment can exceed the authentic biological signal. In this setting, sequencing outputs may reflect background contaminants rather than resident organisms, which can generate misleading taxa lists and inflated diversity estimates. The absence of consistent contamination controls and incomplete reporting in earlier studies has therefore limited confidence in several published findings [131].

Methodological stringency should be treated as a prerequisite for inference in low-biomass niches. Study protocols should incorporate extraction blanks and reagent-only controls processed in parallel with clinical specimens to quantify background signal and enable contaminant identification. Mock community standards (including dilution series where feasible) should be included to define limits of detection, benchmark laboratory performance, and validate decontamination workflows. The randomization of cases and controls across extraction and sequencing batches is required to prevent batch effects from masquerading as biological differences. Biomass quantification should be reported for every sample using measures such as microbial load estimates or total DNA yield, because extremely low input should trigger heightened scrutiny and conservative interpretation. Bioinformatic pipelines should be preregistered where feasible, and all contaminant filtering steps should be described in sufficient detail to permit replication, including the criteria used to flag taxa as contaminants and the handling of low-abundance features. The clear separation of biological signal from background noise is essential in these niches, and conclusions should remain proportionate to the strength of contamination control. Adherence to these practices will reduce artefactual signals and improve reproducibility, enabling microbiome findings in semen and testicular samples to be interpreted with the level of confidence required for mechanistic claims and clinical translation [132,133].

### 7.5. Mechanistic Dissection of Gut-to-Gonad Signaling

Associations between gut dysbiosis and male-infertility-related phenotypes have been reported, yet the causal pathways connecting intestinal microbial states to testicular and accessory gland function remain insufficiently defined. The mechanisms by which intestinal signals influence the testis, epididymis, prostate, and seminal vesicles are not clearly delineated, and the relative contribution of systemic mediators compared with local reproductive tract signaling has not been resolved. In particular, the extent to which dysbiosis-driven inflammation and metabolite shifts impair BTB integrity and thereby alter the intratesticular milieu remains uncertain [134,135].

Mechanistic progress requires experimental systems that permit causal testing and pathway-level mapping. Gnotobiotic and germ-free animal models can clarify whether specific microbial communities are necessary for normal spermatogenesis, steroidogenesis, and hypothalamic pituitary gonadal axis function, while fecal microbiota transfer and defined consortium reconstitution can establish directionality and specificity of effects. Complementary human-relevant platforms, including organoids and ex vivo cultures of testis, epididymis, and prostate, can be used to interrogate cell-type-specific responses to candidate microbial metabolites and inflammatory mediators under controlled conditions. BTB biology should be incorporated as a central readout through quantitative assessment of tight junction architecture, barrier permeability, and immune cell trafficking in dysbiosis and after microbiome restoration. Parallel profiling of circulating and seminal metabolite signatures can then be linked to tissue responses to identify the mediators most likely to convey gut-derived signals to the gonad. Particular priority should be given to microbial metabolite classes with established host signaling capacity, including short-chain fatty acids, bile acids, and tryptophan-derived indoles, with systematic evaluation of their receptors and downstream pathways in reproductive tissues. A mechanistic framework that traces the sequence from microbial community structure to metabolite flux to receptor engagement and tissue-level response is required to convert correlations into actionable targets for intervention [14].

### 7.6. Paternal Microbiome and Offspring Health

Maternal health and the maternal microbiome are recognized determinants of offspring development, whereas the contribution of the paternal microbiome remains largely speculative in human populations. The existing evidence is dominated by indirect associations and animal studies, leaving uncertainty as to whether paternal microbial states shape sperm epigenetic programming in ways that influence embryonic development, pregnancy course, or early life health. This gap limits the interpretation of emerging mechanistic hypotheses and constrains responsible translation into preconception counseling [93].

Progress requires prospective human preconception cohorts that enroll couples before conception and collect repeated paternal microbiome profiles from stool and semen, together with robust measures of sperm epigenetic state, including DNA methylation signatures and the small RNA payload. Follow-up through pregnancy and early childhood should capture standardized outcomes that reflect fetal growth, birth metrics, neonatal morbidity, and early developmental trajectories while accounting for key maternal and environmental covariates. Such designs would allow evaluation of whether paternal microbiome features track with germline molecular changes and whether these signals predict offspring outcomes. Animal data provide proof of concept, but human confirmation will require harmonized protocols, long-term follow-up, and cautious interpretation. If replicated, the findings would justify testing targeted preconception interventions that improve paternal microbiome health through dietary optimization and other evidence-based strategies, with sperm molecular endpoints and offspring outcomes incorporated as prespecified trial measures [93].

### 7.7. Diagnostic Development

Associations between seminal microbial profiles and male infertility have been reported, yet no microbiome-based diagnostic assay has reached clinical validation. Progress is limited by variability in specimen collection and handling, differences in laboratory workflows, and inconsistent analytical pipelines, all of which contribute to poor reproducibility across studies. In addition, it remains uncertain whether microbiome information improves prediction beyond conventional semen analysis, since many proposed dysbiosis indices and candidate biomarkers have not been tested in adequately powered external cohorts or assessed for clinical utility [136].

Development of a clinically useful assay requires a staged translational pathway with prespecified performance criteria. The first requirement is harmonization of protocols for collection, processing, storage, and nucleic acid extraction, together with standardized sequencing and quality control workflows to reduce site and batch effects. Analytical strategies should prioritize strain-level resolution and incorporate approaches that distinguish viable organisms from residual DNA, so that inferred signatures reflect biologically plausible exposures. Candidate microbial markers or composite microbiome signatures should then be evaluated in independent cohorts and across multiple laboratories using locked models and predefined thresholds. Demonstration of incremental value over standard semen analysis is essential and should be quantified using measures of discrimination and calibration. Only signatures that are reproducible across sites and add clinically meaningful information beyond existing workups should progress toward implementation. If these criteria are met, a practical diagnostic framework could combine seminal microbial profiling with a focused panel of inflammatory or oxidative biomarkers and apply a validated algorithm to generate an interpretable risk estimate for infertility-related phenotypes or assisted reproduction prognosis. Achieving this level of validation will require multicenter collaboration, consistent reporting standards, and transparent benchmarking to ensure that microbiome analytics provide reproducible and clinically actionable information [36,137].

### 7.8. Therapeutic Innovation and Antibiotic Stewardship

Microbiome-directed treatment strategies in male infertility remain limited and are supported by an uneven evidence base. Empiric antibiotic use persists in clinical practice for findings such as leukocytospermia or culture positivity without clear symptoms, yet the fertility benefit is uncertain and broad-spectrum exposure may disrupt commensal communities with potential downstream effects on inflammatory tone, oxidative stress, and sperm quality. Probiotic trials have reported variable results and are frequently constrained by small sample sizes, heterogeneous formulations, and short follow-up, leaving uncertainty about the most effective targets, doses, and patient phenotypes [138].

Therapeutic development should therefore shift toward rationally designed and mechanism-aligned interventions. Defined microbial consortia selected on functional criteria may offer more predictable restoration of dysbiotic states than single-strain preparations. Synbiotic strategies that pair organisms with specific substrates can enhance engraftment and metabolic output, improving the likelihood of sustained biological effects. Postbiotic approaches that deliver purified microbial-derived metabolites or structural components may provide a more controlled method to modulate oxidative and inflammatory pathways within the male reproductive tract while avoiding uncertainties related to live organism persistence. These approaches remain investigational and require staged preclinical validation before fertility-focused trials. Phage-based strategies represent another precision option with the potential to reduce dominant pathobionts in semen through targeted bacterial depletion while limiting collateral disruption of beneficial taxa [139].

Each candidate therapy requires a staged evaluation that progresses from in vitro systems and animal models to phased clinical trials with prespecified safety and efficacy endpoints. Early studies should define dose–response relationships, off-target immunologic effects, and impacts on microbial ecology, while later trials should incorporate clinically meaningful endpoints beyond semen parameters, including time to pregnancy and assisted reproduction outcomes where feasible. Safety monitoring should include systematic adverse event capture and surveillance for unintended shifts in microbial composition and function, particularly for interventions that alter niche competition or bacterial lysis dynamics [11].

In parallel, antibiotic stewardship should be integrated into fertility-focused infection management research. Rather than defaulting to broad-spectrum empiric regimens, comparative trials should test targeted, time-limited therapy for confirmed clinically relevant infections against conservative strategies in low-symptom settings, including observation with structured follow-up or adjunctive anti-inflammatory and microbiome-sparing measures. Such designs can clarify whether antibiotics improve fertility outcomes in specific phenotypes or whether benefits are limited to laboratory surrogates while pregnancy-related outcomes remain unchanged. A stewardship framework that balances eradication of pathogenic burden with preservation of commensal communities is likely to be essential for responsible translation of microbiome-based therapeutics in male infertility [138].

### 7.9. The “Testicular Microbiome” Question

Reports of bacterial DNA in testicular tissue have raised the hypothesis of a resident testicular microbiome, yet the evidence remains insufficient to support this conclusion. The testis represents an exceptionally low-biomass environment in which reagent and environmental contamination can readily dominate sequencing outputs. DNA-based detection alone cannot distinguish true colonization from artifact, nor can it establish viability, metabolic activity, or biological relevance. At present, no body of work has demonstrated a reproducible community of live microorganisms within normal testicular tissue together with functional interaction at the tissue level [140].

Progress in this area requires study designs that treat contamination control and proof of viability as non-negotiable criteria. Tissue collection and processing should incorporate extensive negative controls at each step, including field blanks during sampling and extraction blanks during laboratory processing, with explicit reporting of microbial load to contextualize signal strength. Analytical workflows should apply contamination-aware pipelines and provide transparent documentation of all decontamination steps, including the criteria used to flag taxa as likely contaminants. Beyond sequencing, spatial localization is essential. Methods such as in situ hybridization and spatial transcriptomic approaches applied to intact tissue sections can determine whether bacterial signals co-localize with testicular structures rather than appearing diffusely or along surfaces consistent with contamination. Evidence of viability should be required, using complementary approaches that demonstrate transcriptional activity or recoverable organisms, including detection of bacterial RNA signatures and culture attempts where biologically plausible. Only consistent replication across independent laboratories, coupled with spatial mapping and viability evidence, would justify acceptance of a resident testicular microbiome. Until such standards are met, bacterial signals detected in testicular tissue should be interpreted cautiously and framed as provisional, with contamination and transient exposure remaining the most parsimonious explanations [132].

### 7.10. Ethical and Regulatory Considerations

As microbiome research moves closer to clinical use in male infertility, ethical and regulatory oversight becomes central to credibility, safety, and public trust. A primary concern involves quality control for microbiome-based products, particularly probiotic and live biotherapeutic formulations. Independent evaluations have shown frequent discrepancies between label claims and product content, including unlisted organisms and omission of declared strains. Such variability undermines both efficacy and safety and supports the need for manufacturing standards that verify strain identity, viability, dose, and lot consistency, together with systematic screening for contamination and unwanted antimicrobial resistance determinants. Clear regulatory requirements for product characterization and post-marketing surveillance are therefore essential before broad clinical adoption [141,142].

Robust trial governance is equally important. Clinical studies should be prospectively registered, conducted under transparent oversight, and supported by independent monitoring in adequately powered randomized trials. Informed consent must be strengthened for protocols that include metagenomic and host genomic data, since these datasets can carry privacy implications that extend beyond conventional clinical research. Consent processes should specify what biological materials and data are collected; how they will be analyzed, stored, and shared; and the safeguards used to protect confidentiality. Data stewardship should include secure infrastructure, controlled access procedures, and governance frameworks that anticipate secondary use of multi-omics data while respecting participant autonomy [143].

Responsible clinical translation should proceed in parallel. Microbiome tests and treatments should not be incorporated into routine infertility care until analytical validity, clinical validity, and clinical utility have been demonstrated across independent settings. Communication with patients should remain proportionate to the evidence, emphasizing uncertainty where it persists and avoiding overinterpretation of associative findings. Considerations of access and equity are also relevant, since translation that benefits only a narrow subgroup risks widening disparities in fertility care. Professional societies can support responsible implementation by developing guidance on appropriate indications, minimum validation standards, and integration with established diagnostic and therapeutic pathways [144,145].

Collectively, these priorities support a transition from association-driven observations to causality-informed mechanisms and from heterogeneous pilot studies to reproducible interventions anchored to meaningful outcomes, including pregnancy and live birth [36].

## 8. Conclusions

Microbiome dysbiosis is a plausible and potentially modifiable contributor to male infertility. Perturbations across gut, seminal, and urinary niches may converge on established pathways in andrology, including chronic inflammation, oxidative injury to lipids and DNA, endocrine and metabolic disruption, and altered barrier integrity. The androbactome concept is used here as a pragmatic umbrella for microbes detected across the male urogenital tract, while acknowledging that low-biomass sampling requires contamination-aware interpretation and does not imply viable testicular colonization.

Clinical data remain preliminary. Small, randomized trials, interpreted cautiously, report improvements in selected semen parameters and oxidative stress markers in settings such as idiopathic male factor infertility and the early post-varicocelectomy period. These interventions appear safe and biologically plausible as adjuncts to lifestyle optimization and standard care. However, heterogeneity in study design and strain composition, modest sample sizes, significant risk of bias, and limited reporting of pregnancy and live birth outcomes argue against routine clinical use at present. Most available human studies are small, largely single-center, and short-term, and few are powered for pregnancy or live birth outcomes. Accordingly, any observed changes in semen parameters and redox markers should be viewed as intermediate signals rather than patient-relevant endpoints pending larger confirmatory trials.

In current practice, the most defensible near-term application is phenotype-guided adjunctive use with objective monitoring and stopping rules, rather than routine use in unselected men or as a substitute for established evaluation and treatment pathways. Counseling should emphasize the uncertainty regarding effects on pregnancy and live birth and the limited durability data.

Progress depends on rigorous, strain-resolved, contamination-aware study designs and harmonized outcome sets that extend beyond semen analysis to pregnancy and live birth. Future trials should prespecify candidate clinical phenotypes and define actionable monitoring endpoints and stopping criteria to support individualized decision making. Mechanistic studies linking specific microbial functions and metabolites to host immune, redox, and endocrine pathways are required to move from association to causation. If supported by robust human evidence, microbiome-informed diagnostics and targeted interventions may become validated components of infertility care. At present, the clinical signal is promising but preliminary, and routine adoption is premature without adequately powered RCTs assessing pregnancy and live-birth endpoints.

## Figures and Tables

**Table 1:** Database-specific search strategies (executed 31 December 2025).

Database	Platform	Search String	Limits/Filters
PubMed/MEDLINE	PubMed	(“Infertility, Male”[MeSH] OR “male infertil”[tiab] OR “male subfertility”[tiab] OR “semen quality”[tiab] OR “sperm parameter”[tiab] OR “oligozoospermia”[tiab] OR “azoospermia”[tiab]) AND (“Microbiota”[MeSH] OR “microbiome”[tiab] OR “microbiota”[tiab] OR “dysbiosis”[tiab] OR “microbial flora”[tiab] OR “16S rRNA”[tiab] OR “metagenomic”[tiab] OR “Probiotics”[MeSH] OR “probiotic”[tiab] OR “synbiotic”[tiab] OR “prebiotic”[tiab]) AND (English[lang] NOT animals[MeSH])	English; animal-only records excluded
Embase	Elsevier Embase	(‘male infertility’/exp OR ‘male infertility’:ti,ab OR ‘male subfertility’:ti,ab OR ‘semen quality’:ti,ab OR ‘sperm parameter’:ti,ab OR ‘oligozoospermia’:ti,ab OR ‘azoospermia’:ti,ab) AND (‘microbiome’/exp OR microbiome:ti,ab OR microbiota:ti,ab OR microflora:ti,ab OR dysbiosis:ti,ab OR ‘16s ribosomal rna’/exp OR ‘16s rrna’:ti,ab OR metagenom:ti,ab OR ‘probiotic agent’/exp OR probiotic:ti,ab OR synbiotic:ti,ab OR prebiotic:ti,ab) AND [english]/lim AND [humans]/lim	English; Humans
Web of Science	Core Collection	TS=((male infertil OR semen quality OR sperm parameter OR oligozoospermia OR azoospermia) AND (microbiome OR microbiota OR dysbiosis OR microflora OR probiotic OR synbiotic OR prebiotic))	Refined by language: English
Cochrane CENTRAL	Cochrane Library	(male infertil OR semen OR sperm) AND (microbiome OR microbiota OR dysbiosis OR probiotic OR synbiotic OR prebiotic)	No date limits applied in database; English-language records prioritized during screening

Abbreviations: MeSH, Medical Subject Headings; ti,ab, title/abstract; exp, exploded subject heading; TS, topic field; rRNA, ribosomal RNA.

**Table 2:** Domain-level risk-of-bias and methodological quality overview used for interpretive weighting.

Domain	What Was Assessed (Brief Criteria)	Rating Categories/Summary	How It Was Used in Synthesis
Randomized Controlled Trials (Cochrane RoB 2 Domains)			
Randomization process	Sequence generation and allocation concealment; baseline balance; randomization reporting.	Low/Some concerns/High (*n* = 3/3/0)	Unclear methods or randomization flaws reduced confidence in causal inference.
Deviations from intended interventions	Blinding; adherence and co-interventions; intention-to-treat analysis where applicable.	Low/Some concerns/High (*n* = 4/1/1)	Signals were considered exploratory when blinding or adherence was poorly described.
Missing outcome data	Attrition, exclusions, and handling of missing semen/biomarker outcomes.	Low/Some concerns/High (*n* = 4/2/0)	High or uneven attrition down-weighted effect estimates.
Measurement of outcomes	Objectivity and standardization of semen analysis/biomarkers; outcome assessor blinding; timing.	Low/Some concerns/High (*n* = 5/1/0)	Less standardized measurement reduced confidence in between-group differences.
Selection of reported results	Prespecification of endpoints; selective reporting; protocol/registration if available.	Low/Some concerns/High (*n* = 1/5/0)	Selective reporting concerns reduced the weight of conclusions.
Observational and nonrandomized interventional studies (domain-based appraisal)			
Selection bias and phenotype definition	Recruitment methods; eligibility; definition of infertility phenotype and comparators.	Lower/Moderate/Higher risk (*n* = 12/30/19)	Poorly defined phenotypes limited generalizability and interpretability.
Confounding control	Adjustment for age, BMI, smoking, abstinence period, antibiotics, comorbidities, and female-factor context when reported.	Lower/Moderate/Higher risk (*n* = 3/15/43)	Uncontrolled confounding down-weighted causal language.
Exposure/intervention characterization	Microbiome niche and methods; for interventions: strain composition, dose, duration, and co-interventions.	Lower/Moderate/Higher risk (*n* = 10/35/16)	Insufficient characterization reduced comparability across studies.
Outcome definition and ascertainment	Consistency with WHO semen analysis; laboratory methods for DNA fragmentation/oxidative markers; clinical outcomes definition.	Lower/Moderate/Higher risk (*n* = 50/11/0)	Nonstandard outcomes were treated as surrogate signals only.
Reporting completeness and missing data	Transparency of methods/results; handling of missing data; selective outcome reporting.	Lower/Moderate/Higher risk (*n* = 5/35/21)	Incomplete reporting reduced interpretive weight.
Microbiome-specific rigor (low-biomass specimens and analytical quality)			
Contamination control (semen/urine)	Negative controls/blanks; contamination-aware workflows; decontamination reporting.	Adequate/Partial/Not reported (*n* = 1/3/4)	Insufficient contamination control substantially down-weighted findings.
Batch effects and sequencing/biomass reporting	Handling of batch effects; sequencing depth; biomass proxies; reproducibility measures.	Adequate/Partial/Not reported (*n* = 1/5/2)	Unaddressed batch effects reduced confidence in differential abundance signals.
Bioinformatics and statistics	Transparent pipelines; compositionality-aware analyses; multiple-testing control; data availability where possible.	Adequate/Partial/Not reported (*n* = 1/6/1)	Analytical limitations reduced the strength of association claims.
Overall confidence (per evidence cluster)	Integrated judgment across domains, prioritizing study design, reporting completeness, and microbiome-specific rigor.	Higher/Moderate/Lower confidence (assigned per cluster): Probiotic RCTs—Moderate; Male reproductive tract microbiome (semen/urine) observational—Lower; Gut microbiome (observational and MR)—Moderate.	Used to frame conclusions and avoid overstatement; studies were not excluded solely based on appraisal.

Note: Studies were not excluded solely on the basis of appraisal; rather, methodological limitations were used to down-weight confidence in findings where appropriate. Abbreviations: BMI, body mass index; DNA, deoxyribonucleic acid; MR, Mendelian randomization; RCT, randomized controlled trial; RoB 2, Risk of Bias 2; WHO, World Health Organization.

**Table 3:** Randomized controlled trials of probiotic or synbiotic supplementation in men with impaired semen quality.

Study	Design/*n*	Population	Intervention	Primary Endpoint	Pregnancy/Live Birth (Y/N)	Outcomes (WHO → DFI → TAC/MDA → Inflammation → Hormones → AEs)	Major Limitations
Zarehoroki et al., 2025, Iran [108]	DB RCT; *n* = 73	Idiopathic OAT	Synbiotic FamiLact^®^ 500 mg/day + SperiGen vs. SperiGen + placebo 3 mo	WHO progressive motility (3 mo)	N	• WHO: Mot ↑; Immotile ↓; Vol NR; Conc NR; Morph NR • DFI ↓ • TAC/MDA NR • Inflammation NR • Hormones NR • AEs: well tolerated	• Single center • Antioxidant in both arms • 3 mo; no fertility endpoints
Asadi et al., 2023, Iran [17]	DB RCT; *n* = 78	Post-varicocelectomy	Synbiotic FamiLact^®^ 500 mg/day vs. placebo 3 mo post-op	WHO concentration (3 mo)	N	• WHO: Conc ↑; Morph ↑; Mot ns; Vol ns • DFI NR • TAC/MDA NR • Inflammation NR • Hormones NR • AEs: well tolerated	• Single center • Post-operative cohort • 3 mo; no fertility endpoints
Helli et al., 2022, Iran [107]	DB RCT; *n* = 52	Idiopathic OAT	Multi-strain probiotic 2 × 10^11^ CFU/day vs. placebo 10 wk	WHO total motility (10 wk)	N	• WHO: Vol ↑; Conc ↑; Total count ↑; Mot ↑; Morph NR • DFI NR • TAC ↑; MDA ↓ • CRP ↓; TNFα ↓ • FSH/LH/T ns • AEs: well tolerated	• Single center; small *n* • 10 wk; no fertility endpoints
Abbasi et al., 2021, Iran [18]	TB RCT; *n* = 56	Idiopathic infertility (OAT spectrum)	Synbiotic FamiLact^®^ ~10^9^ CFU/day vs. placebo 80 d	DFI (80 d)	N	• WHO: Conc ↑; Mot ↑; Morph ↑; Vol NR • DFI ↓ • Oxidative stress ↓ (lipid peroxidation); TAC/MDA NR • Inflammation NR • Hormones NR • AEs: no serious AEs	• Single center; small *n* • 80 d; heterogeneous phenotypes • No fertility endpoints
Maretti and Cavallini, 2017, Italy [109]	DB RCT; *n* = 41	Idiopathic OAT	Flortec^®^ synbiotic L. paracasei B21060 5 × 10^9^ CFU/day + prebiotics + glutamine vs. placebo 24 wk	WHO progressive motility (24 wk)	N	• WHO: Vol ↑; Conc ↑; Total count ↑; Mot ↑; Morph ↑ • DFI NR • TAC/MDA NR • Inflammation NR • FSH/LH/T ↑ • AEs: good tolerance	• Single center; small *n* • Multi-ingredient formulation • No fertility endpoints
Valcarce et al., 2017, Spain [60]	Randomized cross-over; *n* = 9	Asthenozoospermia	Probiotic L. rhamnosus CECT8361 + B. longum CECT7347 ~10^9^ CFU/day 6 wk + washout	WHO progressive motility (6 wk)	N	• WHO: Mot ↑ (on-treatment); other WHO NR • DFI ↓ (on-treatment) • ROS (H2O2) ↓; TAC/MDA NR • Inflammation NR • Hormones NR • AEs: no safety issues	• Very small *n* • Short periods; washout reversal • No fertility endpoints

Notes: ↑ increase; ↓ decrease; ns no significant difference; NR not reported. Abbreviations: AEs, adverse events; Conc, concentration; CRP, C-reactive protein; DB, double blind; DFI, DNA fragmentation index; MDA, malondialdehyde; Mot, motility; Morph, morphology; OAT, oligoasthenoteratozospermia; RCT, randomized controlled trial; ROS, reactive oxygen species; TAC, total antioxidant capacity; TB, triple blind; T, testosterone; TNFα, tumor necrosis factor alpha; Vol, semen volume.

**Table 4:** Summary matrix of direction of effects across randomized trials on key semen and redox endpoints.

Study	Motility	Concentration	Morphology	DFI	TAC/MDA
Zarehoroki et al., 2025, Iran [108]	↑	NR	NR	↓	NR
Asadi et al., 2023, Iran [17]	↔	↑	↑	NR	NR
Helli et al., 2022, Iran [107]	↑	↑	NR	NR	↑/↓
Abbasi et al., 2021, Iran [18]	↑	↑	↑	↓	↓
Maretti and Cavallini, 2017, Italy [109]	↑	↑	↑	NR	NR
Valcarce et al., 2017, Spain [60]	↑	NR	NR	↓	↓

Notes: ↑ significant improvement/increase; ↓ significant reduction; ↔ no significant difference; NR not reported/not assessed. TAC/MDA: ↑ TAC; ↓ MDA or oxidative marker; ↑/↓ both reported.

## Data Availability

No new data were created or analyzed in this study. Data sharing is not applicable to this article.

## Supplementary Materials

The following supporting information can be downloaded at: https://www.mdpi.com/article/10.3390/jpm16020099/s1, File S1: 2020 PRISMA checklist.
